# 非小细胞肺癌*EGFR*和*ALK*基因双突变研究进展

**DOI:** 10.3779/j.issn.1009-3419.2018.09.07

**Published:** 2018-09-20

**Authors:** 鑫 王, 殿胜 钟

**Affiliations:** 300052 天津，天津医科大学总医院肿瘤内科 Department of Medical Oncology, Tianjin Medical University General Hospital, Tianjin 300052, China

**Keywords:** 肺肿瘤, 表皮生长因子受体, 间变淋巴瘤激酶, Lung neoplasms, Epidermal growth factor receptor (EGFR), Anaplastic lymphoma kinase (ALK)

## Abstract

分子靶向治疗是目前非小细胞肺癌（non-small cell lung cancer, NSCLC）最热门研究的领域之一，表皮生长因子受体（epidermal growth factor receptor, EGFR）和间变淋巴瘤激酶（anaplastic lymphoma kinase, ALK）是NSCLC最重要的两个驱动基因，早期研究认为是独立的分子事件，相互排斥，但不断有*EGFR*和*ALK*双突变的病例或小样本研究报道。*EGFR*和*ALK*双突变作为罕见分子事件，发生率约为1%，其临床病理特点还不很清楚，治疗缺乏定论。本综述汇总已发表病例报道，发现*EGFR*和*ALK*基因双突变多见于女性、东亚、不吸烟、Ⅳ期肺腺癌，一线治疗可选择EGFR或ALK酪氨酸激酶抑制剂（tyrosine kinase inhibitors, TKIs）。然而，目前双突变的起源和耐药机制研究较少，需要更深入的基础和临床研究。

## Abstract (en)

Molecular target therapy is one of the most popular field of non-small cell lung cancer (NSCLC) treatmnet. Epidermal growth factor receptor (*EGFR*) mutation and anaplastic lymphoma kinase (ALK) rearragement are the most important two oncogenic drivers in NSCLC, early studies suggested that *EGFR* mutations and ALK rearrangements are mutually exclusive, but isolated cases or small sample research with concomitant EGFR and ALK alterations have been constantly reported. The co-occurrence of *EGFR* mutations and anaplastic lymphoma kinase (ALK) rearrangements constitutes a rare molecular, the frequency of *EGFR*/*ALK* co-alterations was about 1%, however, little has been known about clinicopathologic feature and treatment. This review summarized published case report, EGFR and ALK alterations are common in female, Asian origin, never smoker, Ⅳ stage, and denocarcinomas. First-line treatment can choose EGFR or ALK tyrosine kinase inhibitors (TKIs). However, studies about the origin and resistance mechanism in EGFR/ALK co-alterations are little, require more experimental and clinical research.

肺癌是我国最常见和病死率最高的恶性肿瘤^[1]^。非小细胞肺癌（non-small cell lung cancer, NSCLC）占肺癌的80%-85%，随着NSCLC发病机制研究的不断深入，驱动基因的发现，靶向治疗显著改善驱动阳性晚期NSCLC的预后。表皮生长因子受体（epidermal growth factor receptor, *EGFR*）突变和间变淋巴瘤激酶（anaplastic lymphoma kinase, *ALK*）重排作为NSCLC最重要的两个驱动基因，在多数情况下被认为是独立的分子事件，相互排斥^[2]^。ALK或EGFR的改变又是EGFR或ALK酪氨酸激酶抑制剂（tyrosine kinase inhibitors, TKIs）耐药机制之一^[3]^。然而，在临床实践中随着检测技术发展，陆续发现伴有*EGFR*突变和*ALK*重排双突变（简称为“双突变”）的患者^[4-6]^。在强调个体化治疗的时代，双突变患者的临床病理特点还不很清楚，EGFR或ALK TKI等药物疗效如何还没有定论。本文总结近年来有关*EGFR*和*ALK*双突变的相关研究结果，阐述双突变发生率、临床病理特点、治疗和生物学机制。

## 1 *EGFR*和*ALK*双突变的发现和发生率

### 1.1 双突变的发现

2008年Koivunen等^[4]^在305例NSCLC中第1次发现1例双突变患者，同时伴有19外显子缺失和棘皮动物微管相关类蛋白4（echinoderm microtubule-associated protein-like4, EML4）-*ALK*重排。2010年Zhang等^[5]^对103例NSCLC行EGFR和ALK检测，报道了中国首例双突变患者，为女性、不吸烟、术后肺腺癌，同时存在19外显子缺失和ALK重排，总生存期超过38个月。然而，上述两项报道均为基因检测方面的研究，未提供双突变患者详细的临床病理特征和治疗预后信息。2010年Tiseo等^[7]^发表第1例双突变的病例报道，男性48岁，否认吸烟，右上肺腺癌伴骨转移，临床分期T1N0M1，同时伴有19外显子缺失和EML4-ALK重排，一线给予吉西他滨联合顺铂治疗6个周期，评效为部分缓解（partial repsonse, PR），无进展生存时间（progressive free survival, PFS）为7个月，因肝内新发病灶和骨转移考虑肿瘤进展，给予厄洛替尼治疗2个月无效后死亡。随后，双突变的病例报道或小样本研究越来越多，在不同的种族或地域均有报道。

### 1.2 双突变的发生率

*EGFR*突变是肺腺癌最常见的驱动基因，东亚和高加索人种的突变率分别在50%和10%左右^[8, 9]^。东亚和高加索人种*ALK*重排发生率约5%^[10]^。对*EGFR*突变或*ALK*重排患者，EGFR或ALK TKIs较标准化疗显著提高有效率和延长PFS。

*EGFR*和*ALK*作为最重要的两个NSCLC驱动基因，一般认为它们是相互排斥的。Gainor等^[2]^曾对1, 683例NSCLC检测*EGFR*、*ALK*和*KRAS*，未发现有这三个基因改变相互重叠的患者。但是，随着基因检测深度和广度的增加，两个及两个以上基因改变的发生率明显增加。肺癌突变联盟^[10]^对1, 007例标本，检测10个肺癌相关的基因，两个及以上基因改变发生率高达2.7%，其中*ALK*和*EGFR*双突变3例，发生率为0.3%，其他多为*PIK3CA*、*MET*扩增等非确定驱动基因改变的重叠。Lee等^[11]^报道韩国肺腺癌*EGFR*和*ALK*双突变发生率为0.9%（4/444）。Yang等^[12]^连续检测中国NSCLC患者977例，共发现*EGFR*和*ALK*双突变患者13例，双突变发生率为1.3%。因此，在NSCLC中双突变属于少见或罕见基因改变事件，发生率0.3%-1.3%^[10, 12]^。

影响双突变发生率的可能原因如下：（1）种族因素，双突变可能存在种族差异，鉴于亚裔患者*EGFR*突变率高于高加索人种，亚裔患者双突变的机会增加，文献报道双突变患者以亚裔居多，最高的双突变发生率发生在亚裔人群^[12]^；（2）检测方式，单基因检测或序贯的检测方式会降低双突变发生率；（3）检测技术，Won等^[13]^纳入1, 445例韩国NSCLC患者，采用直接测序法和FISH发现双突变发生率0.3%（4/1, 445），再对ALK阳性患者进行NGS检测，又发现10例*EGFR*突变，双突变发生率增加至1%。相信随着检测深度和灵敏度的提高，未来双突变发生率会增加；（4）肿瘤分期，双突变的文献报道多发生在晚期转移NSCLC中，可能是疾病发展进程中基因改变不断积累的结果；（5）是否伴有驱动基因改变，在*EGFR*突变和*ALK*重排患者中，双突变发生率分别为3.9%和18.6%^[12]^。

## 2 *EGFR*和*ALK*双突变的临床病理特点

*EGFR*突变和*ALK*重排均多见于不/轻度吸烟的肺腺癌，然而，*EGFR*突变多见于亚裔女性患者，*ALK*重排则无性别种族差异、发病年龄上更年轻、病理上粘液型多见。目前伴有双突变患者研究以病例报道为主，最大样本也仅有14例^[13]^，为了更好了解双突变患者临床病理特征和治疗信息，作者收集2017年10月前发表的双突变病例报道，提取患者临床病理、基因检测和治疗数据，共检索到文献20篇^[7, 11-29]^，其中1篇为来自同一中心的不同报道^[25]^未纳入分析，最终纳入患者66例，见表 1。

**1:** 66例*EGFR*和*ALK*双突变患者的临床病理特征 The clinicopathologic features of 66 patients with co-occurrence of *EGFR* and *ALK* mutation

Authors	*n*	Gender	Age	Ethnicity	Smoking history	Staging	*EGFR* mutation	Frist EGFR/ALK TKI	
								Drug	Repsonse rate
Fan^[14]^	1	F	63	Asian	Non	cT2N0M0	19del	ND	ND
Sweis^[15]^	4	2M, 2F	Median 58.5	Caucasian	2Non, 2Yes	Ⅳ	1 23 polymorphism 1 L861Q, 1 19del, 1L858R	2ed ALK TKI 1 crozitinib, 1 Erlotinib	3PD
Kuo^[16]^	1	F	72	Asian	Non	Ⅳ	19del	1Gefinib	PR
Popat^[17]^	1	F	65	Caucasian	Non	Ⅲ	19del	1Erlotinib	CR
Tiseo^[7]^	1	M	48	Caucasian	Non	Ⅳ	19del	1Erlotinib	PD
Chiari^[18]^	1	F	67	Caucasian	Non	Ⅳ	L858R	1Gefinib	PR
Miyanaga^[19]^	1	F	55	Asian	Non	Ⅳ	19del	1Gefinib	PD
Chen^[20]^	1	M	56	Asian	Yes	Ⅳ	19del	1Erlotinib	SD
Tanaka^[21]^	1	M	39	Asian	Yes	Ⅳ	L858R	1Erlotinib	PD
Santelmo^[22]^	1	F	52	Caucasian	Yes	Ⅲ	19del	1Gefinib	PR
Lee^[11]^	4	1F	73	Asian	Yes	1Ⅳ, 1Ⅲ, 1Ⅱ, 1Ⅰ	2 19del, 1 L858R, 1 L718P	1Gefinib	PD
Zhou^[23]^	1	F	47	Asian	Non	Ⅳ	19del	1Gefinib	PD
Baldi^[24]^	1	M	68	Caucasian	Non	Ⅳ	L858R	1Erlotinib	SD
Zhao^[25]^	1	F	48	Asian	Non	Ⅳ	L861Q	1Erlotinib	SD
Won^[13]^	14	6M, 8F	Median 55.5	Asian	12Non, 2Yes	11Ⅳ, 3Ⅰ	10 19del, 2L858R, 1L861Q, 1E866K	2Gefinib, 2Ceritinib, 4Crozitinib	5PR, 2SD, 1PD
Caliez^[26]^	2	2F	62, 45	Caucasian	1Non, 1Yes	1Ⅳ, 1Ⅲ	2 19del	1Erlotinib, 1Afitinib	SD
Schmid^[27]^	5	3M, 2F	Median 60	Caucasian	2Yes, 3No	Ⅳ	3 19del, 1 L858R, 1 exon20ins	1Crozitinib, 1Alectinib, 3EGFR TKI	2PD, 3PR
Ulivi^[28]^	6	5F, 1M	Median 68	Caucasian	3No, 2Yes	Ⅳ	19del	5Gefinib	1CR, 3PR, 2PD
Yang^[12]^	13	8F, 5M	Median 59	Asian	12Non, 1Yes	11Ⅳ, 2Ⅲ	7 19del, 4 l858R, 1k757R; 1Exon20	3Gefinib, 5Erlotinib, 2Afitinib	8PR, 1SD, 1PC
Thumallapally^[29]^	1	M	72	Caucasian	Yes	Ⅳ	L858R	1Crozitinib	PD
Sasaki^[3]^	6	5F, 1M	Median 62	Asian	5 Non, 1 Yes	5Ⅳ, 1Ⅲ	3G719X, 1L858R, 1 19del, 1R803W	2Gefinib, 2Crozitinib, 1Erlotinib	1PR, 2SD, 1PD
M: male; F: female; PR: partial respones; SD: stable disease; PD: progressive disease; EGFR: epidermal growth factor receptor; ALK: anaplastic lymphoma kinase; TKIs: tyrosine kinase inhibitors; ND: non date.									

66例患者中，以女性患者为主，男女比例为1:2，否认吸烟史患者占72.7%（48/66），亚裔患者占65%（43/66），Ⅳ期患者占78.8%（52/66），除1例为鳞腺癌外其余65例患者均为腺癌。中位年龄60岁（37岁-73岁）。由此可见，双突变患者多见于女性、亚裔、不吸烟、Ⅳ期的肺腺癌。

*EGFR*突变类型方面，19外显子缺失38例（57.6%, 38/66），L858R 14例（21.2%, 14/66），其他少见突变类型共14例（21.2%, 14/66）。双突变患者*EGFR*突变依旧以常见19外显子缺失和L858R为主，但是与*EGFR*突变患者比较，双突变中L858R突变发生率相对偏低为21.1%，而少见突变比例较高达到21.2%。

## 3 *EGFR*和*ALK*双突变的治疗和预后

### 3.1 双突变的靶向治疗

如表 1所示，一线EGFR-TKI治疗28例，接受吉非替尼治疗17例，厄洛替尼治疗9例、阿法替尼2例。疗效为完全缓解（complete response, CR）2例（7.1%, 2/28），PR 14例（50%, 14/28），客观有效率（object responsive rate, ORR）57.1%（16/28），稳定（stable disease, SD）4例（14.2%, 4/28），疾病进展（progressive disease, PD）8例（28.6%, 8/28），2例患者EGFR-TKI治疗肿瘤缩小后完全切除。一线ALK TKI治疗治疗13例，其中接受克唑替尼治疗10例，色瑞替尼治疗2例，艾乐替尼治疗1例。因肺栓塞，2周后死亡1例，失访1例，疗效为PR 7例（64%, 7/11），ORR 81.8%（8/11），SD 2例（18%, 2/11），PD 2例（18%, 2/11）。

双突变患者一线靶向治疗有效率为57.1%-64%，疾病控制率64%-82%，与单驱动基因*EGFR*突变或*ALK*重排接受TKIs治疗疗效略低，但仍高于一线化疗有效率要高^[30]^。Yang等^[12]^报道一代EGFR TKI治疗双突变患者10例，有效率为80%，中位PFS为11.2个月，1例患者一线给予克唑替尼治疗后PR，PFS为15.1个月。因此，双突变患者选择单药靶向治疗是合理的。从异质性角度两类靶向药物同时使用时合理的，但毒性增加、治疗成本提高，且目前没有两类靶向药物同时使用报道。有报道认为^[6]^，一线靶向治疗药物的选择，可以参考检测技术、突变丰度以及EGFR和ALK的磷酸化水平。

### 3.2 双突变的化学治疗

一线化疗患者11例，化疗方案均为含铂双药±贝伐珠单抗，未记录化疗疗效3例。疗效为PR 3例（37.5%, 3/8），SD 4例（33.3%, 3/9），PD 1例（12.5%, 1/8）。双突变患者化疗有效率与文献报道数据基本一致^[30]^。

### 3.3 双突变的预后

Lou等^[6]^报道11例双突变、84例*EGFR*突变和23例ALK阳性患者中位生存时间分别为18.5个月、21.3个月和23.7个月（*P*=0.06），三组间统计上无差异，但是双突变患者生存期在数值上最短，且ALK阳性和双突变两组间生存时间比较有显著差异。肺癌突变联盟数据^[10]^也支持该结果，伴有2个以上基因改变的患者生存期短于无或1个基因改变患者。尽管双突变患者OS数值上短于*EGFR*或*ALK*单突变患者，但经过合理靶向治疗，其预后仍好于单纯接受化疗的患者。

## 4 双突变的生物学机制

### 4.1 双突变的分子假说

目前，有两种假说解释双突变：（1）肿瘤异质性，不同的肿瘤细胞携带的驱动基因不同，肿瘤由携带*EGFR*突变或*ALK*重排的两种癌细胞组成；（2）同一肿瘤细胞携带两种驱动基因，肿瘤由同时携带*EGFR*突变和*ALK*重排的一种癌细胞组成。

肿瘤异质性假说认为双突变是多克隆起源，这类患者不是特殊生物亚型，可能一开始体细胞突变的随意组合，单一靶向治疗只能对部分肿瘤细胞有效，EGFR或ALK TKIs联合治疗可能是肿瘤异质性更佳的治疗方法，但是目前仍没有联合治疗的文献报道，联合治疗的疗效无法评价。

同一肿瘤细胞双突变假说认为双突变位于同一个癌细胞，即单克隆起源。Tiseo^[7]^报道，在1例双突变患者，ALK FISH阳性和阴性细胞均能检测到*EGFR*突变，提示双突变可位于同一细胞或一个癌细胞可同时表达*EGFR*突变蛋白或ALK重排蛋白^[12]^。Cai等^[31]^报道使用激光微切割捕获技术，在肿瘤不同区域，既可以发现双突变癌区域，又发现*EGFR*突变或*ALK*重排区域，说明至少双突变可以存在于同一癌细胞上。在同一个肿瘤出现两个驱动基因，肿瘤发生和进展同时依赖于两个激酶、还是其中一个目前还不清楚。因此，弄清楚两个受体的激活状态对靶向治疗的选择十分关键，ALK或EGFR磷酸化的水平可能对治疗有帮助，Yang等^[12]^报道的2例ALK磷酸化水平高患者接受克唑替尼治疗PR，而2例ALK磷酸化水平低的患者克唑替尼治疗后1例PD、1例SD。

### 4.2 双突变的耐药机制

目前双突变的耐药研究较少，仅限于个案报道^[6]^，1例一线厄洛替尼治疗9个月，疗效PR，进展后厄洛替尼间插化疗治疗8个月，疗效SD。进展后，克唑替尼治疗2个月后再次进展。二次活检发现存在19外显子缺失和T790M点突变，ALK阴性，EGFR磷酸化水平高于ALK磷酸化水平，提示T790M点突变对厄洛替尼耐药，磷酸化水平EGFR高于ALK可能是克唑替尼耐药机制。另外1例厄洛替尼治疗13个月后无症状缓慢进展，继续厄洛替尼7个月，出现明显进展，两处新病灶一处出现T790M突变，一处转化为小细胞肺癌，两处新病灶ALK重排均消失。Lou等^[6]^报道5例EGFR TKI治疗后换用克唑替尼治疗后3例出现PD，1例SD，1例PR，显示EGFR TKI治疗进展后序贯使用ALK-TKI疗效并不理想，提示EGFR-TKI治疗进展后，并不是ALK重排的克隆细胞占有优势，需要二次活检明确耐药机制。

## 5 结语与展望

*EGFR*和*ALK*基因双突变发生率1%左右，发生率可能与种族、检测技术、检测方法和肿瘤分期等因素相关。双突变患者多见于女性、东亚、不吸烟、Ⅳ期肺腺癌，*EGFR*少见突变发生率高。一线单药EGFR或ALK TKIs靶向治疗有效率约60%，一线可考虑选择靶向治疗，化疗可作为后线治疗的选择。双突变的预后可能较单驱动基因突变差，但通过合理靶向治疗后总生存能维持在18个月左右。对双突变的起源和耐药机制研究较少，单克隆起源或多克隆起源假说有待证实，靶向治疗耐药后二次活检对后续治疗选择十分重要。目前许多问题值得思考和研究，双突变患者下游分子通路激活程度、单药治疗还是联合治疗，进展后耐药机制等。这些问题需要更多临床和基础研究来回答，以便给患者提供合理的个体化药物治疗。
